# Characterisation of Paediatric Neuroblastic Tumours by Quantitative Structural and Diffusion-Weighted MRI

**DOI:** 10.3390/jcm13226660

**Published:** 2024-11-06

**Authors:** Domenica Tambasco, Margalit Zlotnik, Sayali Joshi, Rahim Moineddin, Shelley Harris, Anita Villani, David Malkin, Daniel A. Morgenstern, Andrea S. Doria

**Affiliations:** 1 Translational Medicine Program, Peter Gilgan Centre for Research and Learning, The Hospital for Sick Children, Toronto, ON M5G 0A4, Canada; 2 Department of Diagnostic and Interventional Radiology, The Hospital for Sick Children, University of Toronto, Toronto, ON M5G 0A4, Canada; 3 Department of Family and Community Medicine, Dalla Lana School of Public Health, University of Toronto, Toronto, ON M5G 1V7, Canada; 4 Divisions of Epidemiology and Occupational and Environmental Health, Dalla Lana School of Public Health, University of Toronto, Toronto, ON M5T 3M7, Canada; shelley.harris@utoronto.ca; 5 Division of Haematology/Oncology, The Hospital for Sick Children, University of Toronto, Toronto, ON M5G 1X8, Canada; 6 Department of Medical Imaging, University of Toronto, Toronto, ON M5G 0A4, Canada

**Keywords:** diffusion-weighted MRI, apparent diffusion coefficient, tumour volume, neuroblastic tumours, children

## Abstract

**Purpose**: To determine the diagnostic accuracy of quantitative diffusion-weighted (DW) MRI apparent diffusion coefficient (ADC) and tumour volumes to differentiate between malignant (neuroblastoma (NB)) and benign types of neuroblastic tumours (ganglioneuroma (GN) and ganglioneuroblastoma (GNB)) using different region-of-interest (ROI) sizes. **Materials and Methods**: This single-centre retrospective study included malignant and benign paediatric neuroblastic tumours that had undergone DW MRI at diagnosis. The outcome was diagnostic accuracy of the tumour volume from structural and ADC DW MRI, in comparison to histopathology (reference standard). **Results**: Data from 40 patients (NB, *n* = 24; GNB, *n* = 6; GN, *n* = 10), 18 (45%) females and 22 (55%) males, with a median age at diagnosis of 21 months (NB), 64 months (GNB), and 133 months (GN), respectively, ranging from 0 to 193 months, were evaluated. The area under the receiver operating characteristic (AUROC) curve for ADC for discriminating between neuroblastic tumours’ histopathology for a small ROI was 0.86 (95% CI: 0.75–0.98), and for a large ROI, 0.83 (95% CI: 0.71–0.96). An ADC cut-off value of 1.06 × 10^−3^ mm^2^/s was able to distinguish malignant from benign tumours with 83% (68–98%) sensitivity and 75% (95% CI: 54–98%) specificity. Tumour volume was not indicative of malignant vs. benign tumour diagnosis. **Conclusions**: In this study, both small and large ROIs used to derive ADC DW MRI metrics demonstrated high accuracy to differentiate malignant from benign neuroblastic tumours, with the ADC AUROC for the averaged multiple small ROIs being slightly greater than that of large ROIs, but with overlapping 95% CIs. This should be taken into consideration for standardisation of ROI-related data analysis by international initiatives.

## 1. Introduction

Neuroblastic tumours are derived from embryonic neural crest cells of the peripheral sympathetic nervous system and are comprised of neuroblastoma (NB), ganglioneuroblastoma (GNB), and ganglioneuroma (GN). These tumours exhibit varying degrees of maturation, from undifferentiated and aggressive NBs to the more differentiated and benign GNs, and they are diagnosed mainly by histopathology, with the aid of both clinical and imaging modalities [1,2,3,4]. It is important to distinguish between the three forms of neuroblastic tumours, as the management for these forms varies substantially. Whereas neuroblastomas and ganglioneuroblastomas are treated by surgical resection, chemotherapy, or radiotherapy [5,6], ganglioneuromas are monitored or surgically resected.

Currently, conventional imaging cannot reliably differentiate NBs from other neuroblastic tumours, such as GNs or GNBs that occur in the same locations and have similar signal characteristics on MRI, although GN tends to be more homogeneous [1,7,8,9]. Thus, histopathological confirmation is mandatory in a case of suspected NB and can be obtained from a biopsy (bone marrow or primary tumour) or surgical resection of the primary tumour [2,9,10,11]. It is well known that histopathology is strongly correlated with clinical tumour aggressiveness and ultimate outcomes and prognosis [12,13,14,15].

Tumour volume is often considered one of the distinguishing features between malignant and benign tumours. It is generally presumed that larger tumour volumes are related to increased malignant potential [16]. However, calculations of tumour volume assuming a spherical shape may result in either under- or over-estimates of the true volume [17]. Furthermore, few studies have examined this quantifiable MR imaging feature for correlation to the malignancy of neuroblastoma [18].

While structural MRI is useful for mass detection, diffusion-weighted (DW) MRI helps in achieving the diagnosis, providing information regarding tumour grade and type, and monitoring the treatment response. This technique generates signal contrast based on differences in Brownian (random water molecule) motion [19], providing “functional” information about the tumour obtained by probing the free diffusivity of water molecules into intra- and inter-cellular spaces that mainly depend on cellularity in tumours [20]. Although this technique has been used for a long period of time to evaluate tumour diffusion, assessment of region-of-interest (ROI)-related methods to measure tumour changes, in order to reduce measurement errors and bias in the derived imaging metrics, is lacking in the literature. 

Although previous studies have addressed the value of DW MRI in distinguishing benign and malignant neuroblastic tumours, their sample sizes were small, as follows: Gahr et al., N = 15 children (10 neuroblastomas and 6 ganglioneuromas/ganglioneuroblastomas) [21]; Serin et al., N = 24 children (15 neuroblastomas, 4 ganglioneuromas, and 5 ganglioneuroblastomas) [7]; Aslan et al., N = 10 children with neuroblastomas, among other abdominal tumours [22]; Neubauer et al., N = 29 children (19 neuroblastomas, 4 ganglioneuroblastomas, and 6 ganglioneuromas) [9]; Wen et al., N = 25 children (15 neuroblastomas, 7 ganglioneuroblastomas, and 3 ganglioneuromas) [10]; Meeus et al., N = 11 children with neuroblastomas, among other abdominal tumours [23]; Peschmann et al., N = 19 children (15 neuroblastomas, 3 ganglioneuromas, and 1 ganglioneuroblastoma) [24]; Gassenmaier et al., N = 27 children (19 neuroblastomas, 2 ganglioneuromas, and 7 ganglioneuroblastomas) [18]. The above studies have the effect of small-study bias and used arbitrary size selection of ROIs, ranging from small ROIs within the tumour, avoiding cystic and necrotic areas, to large ROIs outlining the entire tumour. It is well known that spatial and size selection of ROIs significantly affects CT and MRI diffusion and perfusion analysis [25,26]. 

To our knowledge, this study is one of the largest studies, if not the largest study, in the literature to assess the accuracy of DW MRI to distinguish different malignant and benign histologic neuroblastic tumour types from the perspective of the ROI method used to derive imaging metrics. This information is essential for utilisation of appropriate post-processing methods in future prospective clinical trials and in clinical practice. So far, few, if any, studies have systematically reported the influence of the size and position of ROI selection on the diagnostic accuracy of DWMRI to distinguish histologic neuroblastic tumour types. The objective of this study was to assess the role of ADCs of DW MRI and tumour volumes in discriminating between benign and malignant neuroblastic tumours of a cohort attending a tertiary paediatric hospital using different region-of-interest (ROI) sizes for data analysis.

## 2. Materials and Methods

This study was conducted in accordance with the Declaration of Helsinki and received approval from The Sick Children’s Hospital Research Ethics Board (REB) office (the date of approval was 08 June 2020, REB#1000067979). Patient consent was waived given the retrospective nature of this study for which obtaining retrospective consent from a large number of patients and families who were no longer followed in our hospital might not be feasible. 

### 2.1. Patients

A master list of consecutive patients who had been diagnosed with neuroblastic tumours (NB, GNB, or GN) and underwent MR imaging examinations in the period from 1 January 2008 to 31 December 2020, in our institution, a tertiary paediatric centre in Canada, was generated from the oncology database and health records, yielding a total of 668 patients. 

The inclusion criteria for participants in this retrospective study were that participants had ages between 0 and 18 years at the time of the imaging assessment, had a confirmed histopathologic diagnosis of NB, GNB, or GN, availability of conventional and DW MR imaging data performed prior to treatment, and availability of clinical data. 

Imaging data were retrieved from our hospital Picture Archiving and Communication System (PACS: 7 SP1.1.0, Centricity Universal Viewer) for further analysis. Demographic, clinical, and pathology data were obtained from electronic medical records (Chartmax and EPIC), and we followed internal guidelines for preserving patients’ identities and confidentiality.

### 2.2. Clinical and Qualitative Imaging Features

Clinical and qualitative imaging features considered in this study included age at diagnosis, sex, presence of metastasis, and contrast enhancement (Supplementary Table S1). 

### 2.3. Imaging Examinations

#### 2.3.1. MRI Data Acquisition

Study patients underwent clinical routine MRI examinations at 1.5 Tesla (Siemens Avanto, Erlangen, Germany (N = 6), Philips Achieva, Best, Netherlands (N = 23), and GE Signa HDxt. Milwaukee, WI (N = 1)) and 3.0 Tesla (Siemens Skyra, Erlangen, Germany (N = 7), and Philips Achieva, Best, Netherlands (N = 3)) in supine position for the initial diagnosis. Standard pre-contrast and post-contrast sequences were obtained as needed for clinical diagnostic workup (e.g., multiplanar T1- and T2-weighted images, and post-gadolinium T1 images if contrast was administered; Supplementary Table S2). DWI was acquired prior to injection of the contrast agent in all cases, except for two (1 GN and 1 GNB), using the following parameters: free-breathing, TR = 1525–8923 ms, TE = 46–124 ms, b-values = 0–1000 s/mm^2^ (4 b-values were obtained in 1 case, 3 b-values were obtained in 25 cases, 2 b-values were obtained in 7 cases, and no information was available regarding the remaining 7 cases), a flip angle of 90° for all cases, a slice thickness ranging from 4 to 7 mm, pixel bandwidth ranging from 1185 to 2774 Hz, matrix ranging between 96 × 92 and 188 × 206, and total scan time ranging between 30 and 126 min. The field-of-view (FOV) was adapted as needed for optimal fit in each patient and ranged from 15 to 45 cm. Mean/median ADC maps (unit: ×10^−6^ mm^2^/s) were automatically calculated on the MRI console immediately upon completion of the examinations.

Depending on the patients’ size and the MRI machine, one or two body array coils were used to cover the anatomy of interest. Head/spine coils were used to image the brain and spine.

#### 2.3.2. MRI Interpretation

The image analysis was performed on a dedicated radiological workstation by a paediatric radiologist with 8 years of experience in radiology (M.Z.). Discussions were had as needed with a paediatric radiologist with more than 20 years of experience after training (A.S.D.), who obtained information about tumour location, size, and volume, internal characteristics, such as necrosis, calcification, heterogeneity, T1- and T2-weighted predominant signal, presence vs. absence of diffusion restriction, type of contrast enhancement, multifocality or multiple body compartmental involvement, as well as imaging-defined risk factors (IDRFs). The IDRF list is a list of twenty features based on the tumour location and adjacent vital structures used for staging and assessing tumour resectability, including vascular encasement, adjacent organ infiltration, intraspinal invasion, and tracheal compression, among others [27,28]. Presence/absence of metastases at the time of initial imaging was also recorded (Supplementary Table S1).

Tumour volume was obtained using the following ellipsoid formula: volume in cubic centimetres (cc) = (π/6) × antero-posterior (depth in cm) × width (in cm) × cranio-caudal (length in cm) [29]. In our study, we used at least two sequences, T2-weighted or contrast-enhanced T1-weighted axial and coronal (or sagittal) images, to measure the tumour volume. Measurements of ADC values were performed by two independent blinded paediatric radiologists, with 8 and 7 years of experience in radiology, respectively, M.Z. and S.J., after a calibration session supervised by an experienced paediatric radiologist (A.S.D.). Discrepancies in the interpretation of categorical data by the junior readers were solved in consensus with the more experienced reader. Final values for the ADC measurements were obtained by averaging the values of the two readers. If the values provided by the readers for a given MRI examination had a discrepancy of more than 20% between them, another round of measurements was performed by the two readers independently to yield the final value. The readers reviewed all MRI examinations of the study blinded to the histopathological report and to each other’s measurements.

**Figure 1:**
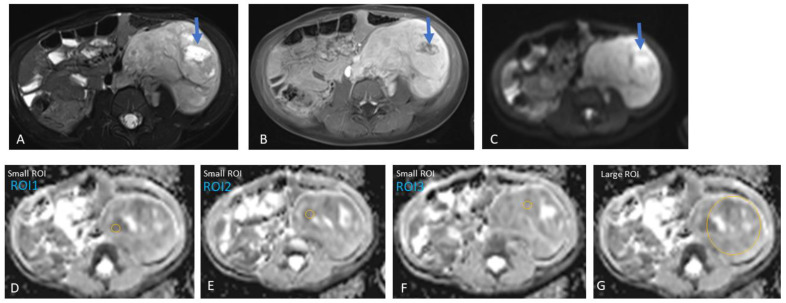
Left retroperitoneal neuroblastoma with areas of necrosis in a three-year-old girl. Heterogeneous tumour, predominantly solid with a focal fluid area (arrow), potentially representing necrosis within the tumour. Therefore, this focal area of fluid was avoided during the placement of the regions-of-interest (ROIs) in the tumour for data analysis. (**A**) Axial T2, (**B**) axial post-gadolinium T1, and (**C**) axial DWI (b-value: 800 mm^2^/s). (**D**–**F**) Three small ROIs on the ADC map with an area of 19.1 mm^2^ and an average value of 1.23 × 10^−3^ mm^2^/s, and (**G**) one large ROI with an average area of 2721.1 mm^2^ and an average value of 1.42 × 10^−3^ mm^2^/s.

For assessment of the ADC values of the primary tumour, two methods were used:

Method 1: For the multiple small ROIs method, three areas of 10–20 mm^2^ each were manually chosen, targeting tumour portions with the highest signal on DWI (using the highest b-value available), corresponding to the lowest ADC value not representing fluid on comparison with the corresponding T1/T2/post-gadolinium T1 images, presumably representing areas with the highest tumour cellularity [9]. Portions of the tumour showing a restricted DWI signal along with a high ADC were avoided due to the potential shine-through effect (Figure 1).

**Figure 2:**
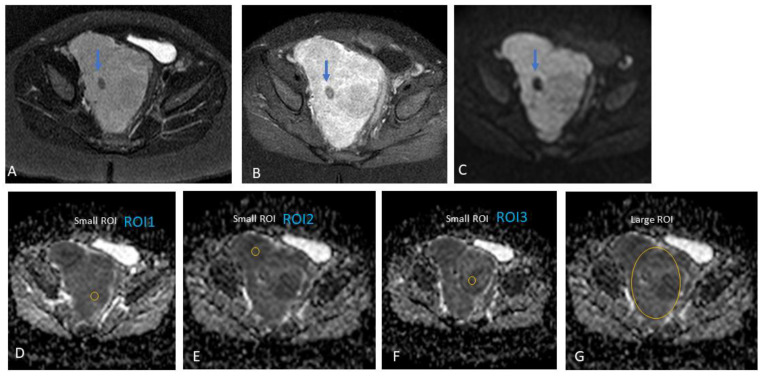
Presacral neuroblastoma in a one-year-old boy. Heterogeneous tumour, predominantly solid. Note should be made of the small focal area of low signal intensity on T2 (**A**), post-contrast T1 (**B**), and diffusion-weighted (DW) trace (**C**) MR images (arrows), which could represent a focal area of calcification or fibrosis. This focal area was avoided during the placement of small regions-of-interest (ROIs) within the tumour during data analysis. (**A**) Axial T2, (**B**) axial post-gadolinium T1, and (**C**) axial DWI (b-value: 600 mm^2^/s). (**D**–**F**) Three small ROIs on the ADC map with an area of 17.6 mm^2^ and an average value of 0.78 × 10^−3^ mm^2^/s, and (**G**) one large ROI with an average area of 1280.2 mm^2^ and an average value of 0.93 × 10^−3^ mm^2^/s.

**Figure 3:**
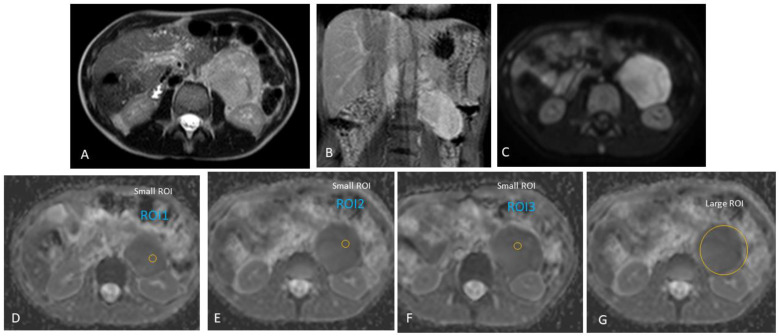
Left retroperitoneal ganglioneuroblastoma in an 11-year-old boy, presenting with an abdominal mass incidentally diagnosed on US. Homogenous solid tumour. Therefore, the regions-of-interest (ROIs) were randomly placed within the tumour during the analysis of apparent diffusion coefficient (ADC) diffusion-weighted (DW) MRI values. (**A**) Axial T2, (**B**) coronal post-gadolinium T1, and (**C**) axial DWI (b-value: 500 mm^2^/s). (**D**–**F**) Three small regions-of-interest (ROIs) on the ADC map with an average area of 15.3 mm^2^; ADC for the small ROI method, 1.05 × 10^−3^ mm^2^/s. (**G**) One large ROI on the ADC map with an average area of 1823.4 mm^2^; ADC for the large ROI method, 1.07 × 10^−3^ mm^2^/s.

**Figure 4:**
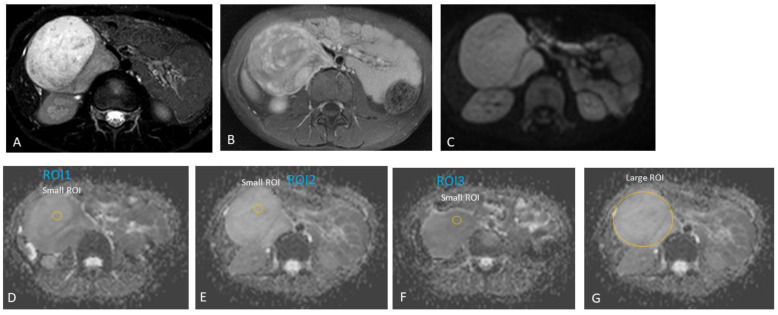
Right retroperitoneal ganglioneuroma in an eight-year-old boy. Incidental finding of an abdominal mass in the right upper quadrant on ultrasound after head injury. Heterogeneous tumour, predominantly solid. Therefore, the regions-of-interest (ROIs) were randomly placed within the tumour during the analysis of apparent diffusion coefficient (ADC) diffusion-weighted (DW) MRI values. (**A**) Axial T2, (**B**) axial post-gadolinium T1, and (**C**) axial DWI (b-value: 600 mm^2^/s). (**D**–**F**) Three small regions-of-interest (ROIs) on the ADC map with an area of 19.1 mm^2^; ADC for the small ROI method, 1.75 × 10^−3^ mm^2^/s. (**G**) One large ROI on the ADC map with an average area of 2990.7 mm^2^; ADC for the large ROI method, 2.55 × 10^−3^ mm^2^/s.

Furthermore, we did not include areas of necrosis, haemorrhage, or calcifications (based on the appearance on T2-weighted and post-contrast T1 images) in our ROIs (Figure 2, Figure 3 and Figure 4). A mean/median ADC value for small ROIs was calculated from the six readings obtained.

Method 2: For the large ROI method, the ROI encompassed the entire tumour at the level of its largest transverse cross-section. The rationale for using the largest diameter was based on other studies using this method [7,9,24]. Similar to the small ROI method, these readings were combined to obtain a mean/median ADC value for large ROIs. Numerical values for the ADC were generated from the PACS software (7 SP1.1.0, Centricity Universal Viewer).

#### 2.3.3. Reference Standard

The reference standard for the study assessing the diagnostic test accuracy was histopathology obtained within one month of the DW MRI scan performance. Exceptions for pathology dates up to four months were considered in cases where there was no significant interval change or treatments received, or the combination of clinical, laboratory, and other imaging results was used to confirm the diagnosis.

#### 2.3.4. Effect Size Related to Available Sample Size

We assumed that the prevalence of a positive test in the control group was 80%. Our study had a total sample size of 40 patients (16 in the ‘control’ and 24 in the ‘case’ group), and with a <0.05 type 1 error rate, we would have 80% power to detect a prevalence difference of 44% or larger between two groups.

### 2.4. Statistical Methods

Baseline and demographic characteristics were summarised using descriptive statistics (mean with standard deviation for continuous, normally distributed variables, and median with range for non-normally distributed variables, as appropriate).

Two-sided T-tests were used to compare mean ADC values between malignant and benign neuroblastic tumours, and analysis of variance (ANOVA) to compare mean ADC values among NB, GNB, and GN. The Wilcoxon test was used to compare age at diagnosis (as well as tumour volume) between malignant and benign neuroblastic tumours, and the Kruskal–Wallis test to compare age at diagnosis (as well as tumour volume) among NB, GNB, and GN. A Chi-square test was performed for assessing associations between clinical and imaging categorical data, and Fisher’s exact test was used in instances where there were fewer than 5 expected cases per category.

Plots for the area under the receiver operating characteristic curve (AUROC) were generated from the binomial logistic regression model. Confidence intervals (95%) around estimates of accuracy were calculated. An ADC cut-off value—determined by Youden’s Index and corresponding to the peak of the receiver operating characteristic (ROC) curve—was calculated for the small region of interest (ROI) and large region of interest. Similarly, a cut-off value was calculated for total tumour volume. As a basis for comparison, a known clinical feature of severity (age at diagnosis) was also analysed for measures of accuracy.

Cross-tabulations were performed to determine the diagnostic accuracy measures, and logistic regression was performed to calculate the predicted probabilities. The main outcome variable was the severity of the tumour (malignant versus benign). Multinomial logistic regression was performed using the histological type (NB, GNB, and GN) as the outcome variable. We used the backward selection procedure to test which variable(s), if any, could be removed from the model without losing a significant effect. In this case, we chose the 0.1 significance level. We also conducted logistic regression analysis using several categorical (i.e., sex and tumour location) predictor variables in the model to visualise trends in effects. The Hosmer and Lemeshow goodness-of-fit test was performed to assess the diagnostic model fit.

Inter-rater agreement between ADC values according to the multiple small and large ROIs was calculated using the kappa statistic, as well as a percentage concordance over the average values. The kappa coefficient cut-off values used were as follows: ≤0.20 (poor agreement), 0.21–0.40 (fair agreement), 0.41–0.60 (moderate agreement), 0.61–0.80 (substantial agreement), and 0.81–1.00 (excellent agreement) [30].

Statistical analysis was performed using SAS^®^ studio, version 6.0. MedCalc was used to calculate the diagnostic odds ratio (DOR) and logarithmic (log) DOR. Values with an alpha < 0.05 were considered statistically significant.

## 3. Results

Out of the 668 cases retrieved through the databases of this study, 409 (61%) did not have an MRI examination at the time of diagnosis, and 208 (31%) of these did not have DWI. From the 51 (8%) cases that did have DWI, 11 were excluded due to either a long interval between DWI and histopathology or inadequate imaging. Two exceptions for inclusion into the study were made for cases in which there was no interval change or treatment received up to four months of time elapsed between imaging and histopathology. Based on the study criteria, only 40 (6%) of the total cases initially retrieved were eligible for inclusion in the study. Our study population consisted of 24 malignant cases (all with NB, no cases with GNB-N) and 16 benign cases (10 with GN, and 6 with GNB-I). Table 1, Table 2 and Table 3 provides descriptive characteristics of the cohort patients and respective tumours.

The median patient age at diagnosis was 55 months, ranging from 0 to 193 months, with a median age of 96 months (range: 14–193 months) for benign tumours and 10 months (range: 0–119 months) for malignant tumours (*p* < 0.0001). By tumour type, the median age was 132 months (range: 34–193 months) for GN, 58 months (range: 14–140 months) for GNB, and 10 months (range: 0–119 months) for NB (*p* < 0.0001; Table 4). In this study, we used a cut-off of 48 months of age for differentiation of benign and malignant tumours rather than a lower age cut-off, as it would be expected for indication of neuroblastoma prognosis based on the age characteristics of the patient cohort, since we considered a single age cut-off to encompass both patients with benign and malignant tumours.

A summary of the clinical and qualitative imaging features of tumours of the patient cohort is provided in Table 5 and in Supplementary Table S2.

**Figure 5:**
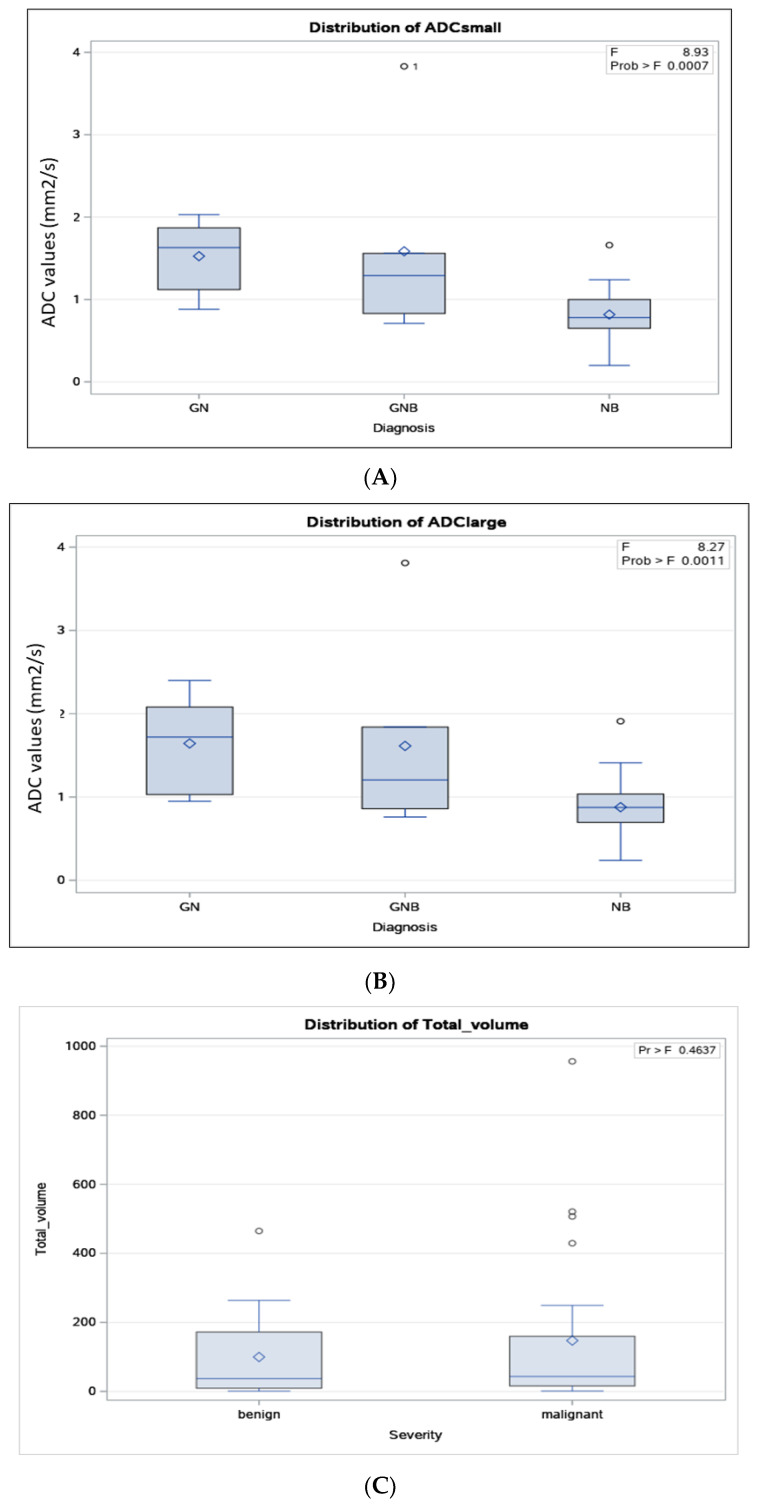

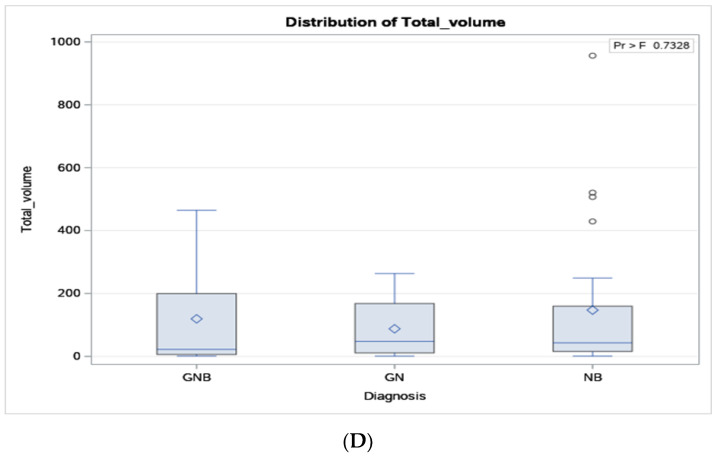
Distribution of apparent diffusion coefficient (ADC) values according to the histopathologic results of the neuroblastic tumours for the small (**A**) and large (**B**) regions-of-interest (ROIs) method. Distribution of tumour volume according to benignity vs. malignancy of the neuroblastic tumours (**C**) and according to histopathologic results (**D**).

The distribution of ADC values according to the histopathologic results of the neuroblastic tumours for small (Figure 5A) and large (Figure 5B) ROIs, and of total tumour volume according to benignity vs. malignancy (Figure 5C) and according to the tumoral histopathology (Figure 5D), are shown in Figure 5. Note should be made to the fact that there were 4 benign cases (25%, out of 16) and 4 malignant cases (17%, out of 24) whose ADC values for small ROIs fell below or above, respectively, the cut-off value.

According to the small ROI method, the median ADC values for malignant and benign neuroblastic tumours were 0.78 × 10^−3^ mm^2^/s (range: 0.2 × 10^−3^–1.92 × 10^−3^, 95% CI: 0.68 × 10^−3^–0.95 × 10^−3^) and 1.53 × 10 ^−3^ mm^2^/s (range: 0.71 × 10^−3^–3.82 × 10^−3^, 95% CI: 1.15 × 10^−3^–1.94 × 10^−3^), respectively. By tumour type, the median ADC value for NB was 0.78 × 10^−3^ mm^2^/s (range: 0.20 × 10^−3^–1.66 × 10^−3^), for GNB-I was 1.29 × 10^−3^ mm^2^/s (range: 0.71 × 10^−3^–3.83 × 10^−3^), and for GN was 1.63 × 10^−3^ mm^2^/s (range: 0.88–2.16 × 10^−3^; Figure 5A; Table 1). The median ADC values for small ROIs for the three groups were different (*p* = 0.0007; Figure 5A; Table 1).

According to the large ROI method, the median ADC values for malignant and benign neuroblastic tumours were 0.88 × 10^−3^ mm^2^/s (range: 0.76 × 10^−3^–1.94 × 10^−3^, 95% CI: 0.73 × 10^−3^–1.03 × 10^−3^) and 1.52 × 10^−3^ mm^2^/s (range: 0.76 × 10^−3^–3.81 × 10^−3^, 95% CI: 1.21 × 10^−3^–2.05 × 10^−3^), respectively. By tumour type, the median ADC for NB was 0.88 × 10^−3^ mm^2^/s (range: 0.24 × 10^−3^–1.66 × 10^−3^), for GNB-I was 1.21 × 10^−3^ mm^2^/s (range: 0.76 × 10^−3^–3.81 × 10^−3^), and for GN was 1.72 × 10^−3^ mm^2^/s (range: 0.95 × 10^−3^–2.40 × 10^−3^; Figure 5B; Table 1). The median ADC values for large ROIs for the three groups were different (*p* = 0.0011; Figure 5B; Table 1).

Tumour volume, on the other hand, was not indicative of malignant vs. benign neuroblastic tumour diagnosis in our study cohort. Tumour volume ranged from 1 cc to 956 cc in our study cohort, with median volumes of 37 cc (range: 1–465 cc) for benign tumours and 43 cc (range: 1–956 cc) for malignant tumours (*p* = 0.46; Figure 5C; Table 1). The median volume was 48 cc (range: 1–264 cc) for GN, 22 cc (range: 1–465) for GNB, and 43 cc (range: 1–956) for NB (Figure 5D; Table 1). However, these tumour volumes were not statistically different based on non-parametric tests (*p* = 0.79).

**Figure 6:**
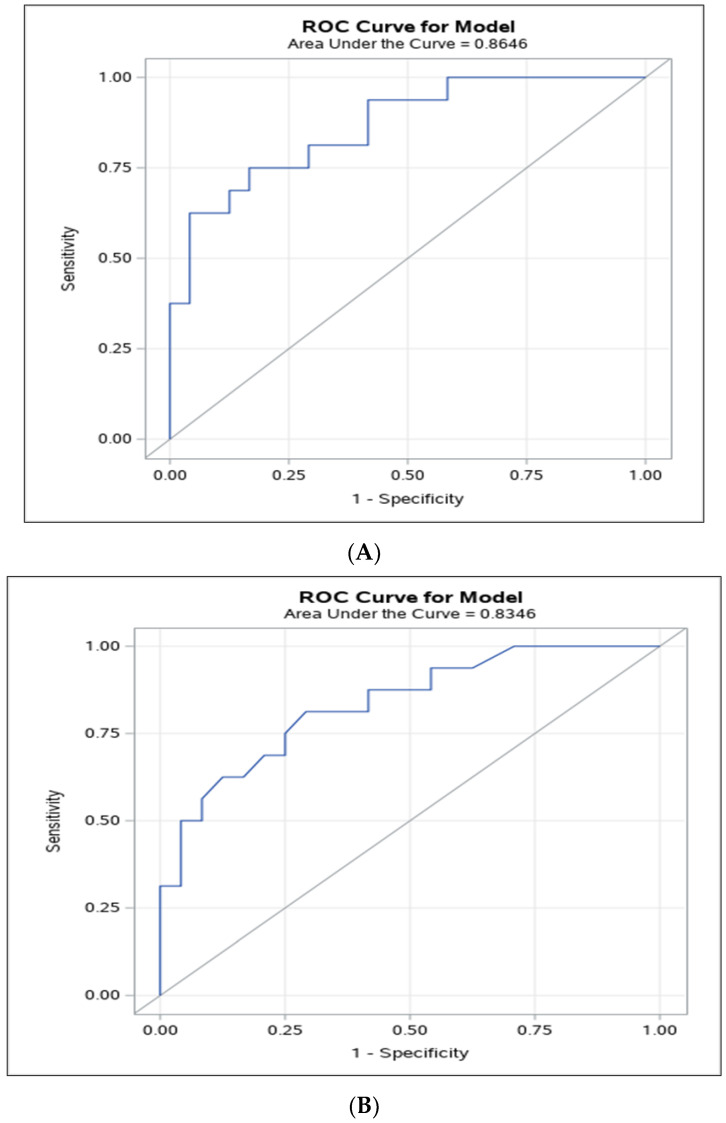
Area under the receiver operating characteristic (AUROC) curve for apparent diffusion coefficient (ADC) cut-off values to predict malignant neuroblastic tumours for the small (**A**) and large (**B**) regions-of-interest method.

Our study results showed an AUC-ROC of 0.86 (95% CI: 0.75–0.98) for discriminating between different neuroblastic tumours’ histopathology with the small ROI method (Figure 6A), and of 0.83 (95% CI: 0.71–0.96) with the large ROI method (Figure 6B). ADC cut-off values of 1.06 × 10^−3^ mm^2^/s and 1.22 × 10^−3^ mm^2^/s were able to differentiate malignant from benign neuroblastic tumours for the small and large ROI methods, respectively. The sensitivity and specificity for distinguishing malignant from benign neuroblastic tumours at this optimal point were 83% (95% CI: 68–98, *p* = 0.001) and 75% (95% CI: 54–96, *p* = 0.046) for the small ROI method, and 79% (95% CI: 63–94, *p* = 0.003) and 83% (95% CI: 62–100, *p* = 0.021) for the large ROI method.

Additional diagnostic test performance measures, quantitative MRI features, and age at diagnosis are available in Table 6.

In the single-variable logistic regression analysis, we used the response variable ‘severity’ with probability modelled ‘benign’, as we expected that the larger the ADC value for the large ROI, the more likely the tumour would be benign. The odds ratio for ADC to predict tumoral benignity was 48.52 (95% Wald confidence limits: 4.09–575.36) using the small ROI method and 21.58 (95% Wald confidence limits: 2.63–177.01) using the large ROI method. In the age-adjusted model, the odds of observing a benign tumour for each unit increase in ADC (for instance, an increase from 0.67 × 10^−3^ to 1.67 × 10^−3^) were 12.19 times greater than observing a malignant tumour for the small ROI method and 5.38 times greater than observing a malignant tumour for the large ROI method (Table 7).

Tests for single-variable associations between diagnosis and clinical features, as well as other imaging variables, showed no association with malignity, except for age at diagnosis and the presence of metastases (*p* = 0.01; Supplementary Table S1).

There was substantial inter-rater agreement (kappa = 0.74, 95% CI: 0.72–0.76) in the ADC values obtained from the pre-established area size for the small ROI (1–2 mm^2^). There was no pre-established area size for the large ROI, which was reflected in the fair agreement (kappa = 0.40, 95% CI: 0.38–0.42) in the manually chosen large area. Despite the variation in the large area chosen, there was good inter-rater agreement (kappa = 0.88, 95% CI: 0.87–0.89) in the actual ADC values obtained using the large ROI method (Supplementary Table S3).

## 4. Discussion

The results of this study indicate a high diagnostic accuracy of ADC values for discriminating between different neuroblastic tumours’ histopathology for both small and large ROI methods, suggesting that ADC DWI MRI has the potential to differentiate malignant from benign neuroblastic tumours regardless of the size of the ROI used for drawing imaging information for ADC data analysis. This MR imaging technique can definitely assist in guiding clinical decisions for management of neuroblastic tumours. Characteristics of our study cases, such as tumour type, age, and sex, were similar to the neuroblastic tumour population of other studies [1,31], suggesting that our results are likely generalisable to other neuroblastic tumour populations. Similarly, our main index test feature (ADC value) and its associated diagnostic accuracy measures aligned with the results obtained in other studies [9,10,21,22,23,24] and with the pooled results of our systematic review and meta-analysis [32].

Our results pointed towards a high diagnostic accuracy of ADC to discriminate between different neuroblastic tumours’ histopathology for both small and large ROI methods. In our study, although numerically the AUROC for the small ROI was slightly larger than the AUROC for the large ROI, there was overlap of 95% CIs of the two methods, failing to show a significant difference. In a multivariate logistic regression analysis, Yang S. et al. [33] showed an AUC of 0.96, with an overall accuracy, sensitivity, and specificity of 93.4%, 96.3%, and 83.8%, respectively, for four clinical and biological predictors (age ≤ 49 months, primary site of tumour adrenal and thoracic, NSE level ≥ 33 ng/mL, and tumour encasing blood vessel) to predict the diagnosis of malignant peripheral neuroblastic tumours (PNTs) [33]. However, while this study considered the largest tumour diameter in its model, it did not consider other quantitative imaging features, such as ADC or total tumour volume. Aslan M. et al., on the other hand, using a ROI of 0.2 cm^2^ placed on the ADC maps that displayed the maximum contrast enhancement on contrast-enhanced T1-weighted imaging, obtained a cut-off value of ≤0.645 × 10^−3^ mm^2^/s to differentiate neuroblastoma from Wilms’ tumour through an ROC curve analysis [22].

Other studies also showed a large AUROC for ADC to differentiate neuroblastic tumours. Peschmann et al. showed that the baseline ADC values were helpful for non-invasive prediction of tumour histopathologic severity, with the mean ADC being significantly lower in NB compared to GNB/GN (0.76 ± 0.11 versus 1.47 ± 0.23 × 10^−3^ mm^2^/s; *p* = 0.003) [24]. Their ROC analysis identified a cut-off value for mean ADC of 1.05 × 10^−3^ mm^2^/s to distinguish between malignant (NB and GNB) and non-malignant neuroblastic tumours (GN), with an AUC of 0.96 (standard error of 0.047 and 95% CI ranging from 0.87 to 1.00) [24]. Serin et al. showed an AUC of 0.80 (95% CI: 0.62–0.98) in the ROC curve analysis to distinguish malignant from benign neuroblastic tumours, with an ADC cut-off value of 0.93 × 10^−3^ mm^2^/s [7]. Different methodologies using quantitative MR diffusion have been proposed to distinguish malignant from benign neuroblastic tumours [34].

Concerning the single-variable logistic regression analysis of this study, the odds ratio for ADC to predict tumoral benignity was very high for the small ROI method (48.52) and high for the large ROI method (21.58). This means that for every one-unit increase in the ADC value, there was an increased odds of 48.52 that the tumour was benign (*p* = 0.002) for the small ROI method and an increased odds of 21.58 that the tumour was benign (*p* = 0.004) for the large ROI method.

An important consideration of our findings, similar to the findings of the aforementioned studies [9,10,14,15,16,17,18,19,20,21,22], is the existence of outliers in the distribution of ADC values according to the histopathologic diagnosis of the neuroblastic tumours (Figure 5A,B). Therefore, the study results suggest that the ADC quantitative measure should not be used in isolation to make a definitive diagnosis but should rather be used as a complementary predictive diagnostic tool, in conjunction with other clinical and laboratory diagnostic tools.

In our study population, the total tumour volume did not appear to be associated with the malignancy histopathologic diagnosis of neuroblastic tumours. There are studies in the literature that have investigated the tumour location and other descriptive characteristics that discriminate neuroblastic tumours [33,35,36,37] and concluded that changes in ADC values precede the neuroblastoma volume reduction post-treatment [38]. To our knowledge, no prior studies have specifically addressed the tumour volume at diagnosis, and only a few studies have examined the tumour volume as a prognostic indicator in the post-chemotherapy response [16,18]. We hypothesise that the lack of association between tumour volume on MRI and either benignity vs. malignancy or tumour histologic category (Figure 5C,D) may, in part, be a result of the older age (and naturally increased body and tumour size) of benign characteristics of tumours at diagnosis. Thus, rather than comparing tumour sizes between malignant and benign cases, it may be more appropriate to compare tumour volumes as a proportion of the patient’s body size. Nevertheless, given this relatively low number of prior studies on the value of tumour volume in differentiating histopathologic categories of neuroblastic tumours and the results of our single-site study, results from larger series are needed. Both size and location can have a significant impact on the management of both malignant and benign neuroblastic tumours, as recognised by imaging guidelines for imaging-defined risk factors [16].

A satisfactory inter-rater agreement was noted in our study, similar or inferior to the results of a previous study that assessed the value of DW MRI in the diagnosis of neuroblastic tumours, which reported coefficients of variation ranging from 5.5% to 7.8% for large and small ROIs [9], and ICCs of up to 0.96, indicating excellent inter-rater agreement for small ROIs [22]. Previous studies have pointed out that placing a single ROI on a representative tumour image can lead to sampling bias; hence, it would not provide an accurate representation of the tumour heterogeneity, as discussed by Bharwani et al. in endometrial cancer [39]. Nougaret et al. [40] argued that a whole-tumour (large) ROI approach compared to single-slice ROI analysis would reduce the sampling bias, thus improving the interobserver agreement. One should note that if a large ROI is used on a single slice, it would include undesirable areas of the tumour for data analysis, such as those containing necrosis/cystic changes. Although in this study both small and large ROIs used to derive ADC DW MRI metrics demonstrated high accuracy to differentiate malignant from benign neuroblastic tumours, with overlapping 95% CIs, numerically, the AUROC of ADC for the small ROI was slightly greater than that of the large ROI (Figure 6A,B), possibly accounting for undesirable areas of necrosis/cystic changes being included in the large ROI sample. This information should be taken into consideration, as guidelines for standardisation of ADC DW MRI data analysis are developed according to a pre-established ROI size and location in the tumour, and they are key for the successful translation of this technique in future clinical trials and clinical practice.

Our study has limitations. As the ADC measurements were obtained by two blinded independent readers, other variables, such as tumour volume and IDRFs, were obtained by one reader supervised by a more experienced reader. Different MRI scanners and techniques were used for the imaging examinations within the study timespan, which may have affected the quality of MR images available and could have a small effect on the variability of measurements. Further, the proposed methods for measuring ROIs in tumours pose challenges, as they are time-consuming and require experience and knowledge about different MRI sequences, yielding a challenge for implementation of a standardised reading protocol, which may be reduced with the development and validation of artificial intelligence methods to ease the process. Studies in other abdominal tumours have shown that the differentiation of benign and malignant lesions based only on ADC quantification may not be straightforward due to the wide overlap of ADC values, such as in pancreatic cancer [41]. Furthermore, although current standard practice in most centres around the globe recognises the use of whole-tumour segmentation for comprehensive quantitative information about the random Brownian motion of water molecules within a voxel of tissue in solid tumours [42], from the research perspective, there is no standard towards the choice of ROIs when measuring ADC maps. In solid tumours other than neuroblastic tumours, ADC maps obtained from spot-measurement methods have been compared with those from whole-tumour methods for differentiation of malignant and benign breast [43] and anterior mediastinal tumours [44].

Future studies comparing IVIM-DW MRI, i.e., perfusion fraction and fast component of diffusion, with conventional DW MRI are suggested, as consistent results using IVIM-DW MRI have been described in solid abdominal tumours, particularly in solid pancreatic lesions [41]. Other promising techniques that may add to the current quantitative ROI-based ADC MRI methods available are quantitative semi-automated DWI volumetry, which can provide an integrated analysis of tissue characteristics by means of automatically calculated ADC values of the whole tumour, as well as an ADC heatmap [18], and intravoxel incoherent motion-derived histogram metrics [40].

In conclusion, our study reaffirmed the high diagnostic accuracy of quantitative ADC DW MRI to non-invasively differentiate malignant from benign neuroblastic tumours with the utilisation of either a large or a small ROI for data analysis. Notably, the AUROC of ADC for averaged, multiple, small ROIs was greater than that of the large ROIs, indicating potential inclusion of undesirable tumour areas in the latter. This highlights the challenge of using large ROIs in heterogeneous tumours, which may compromise ADC analysis. Standardising the ADC DW MRI methodology, including ROI size and location definitions, is crucial for enabling comparative assessment in future clinical trials. Such standardisation could significantly enhance the post-processing analysis of this imaging technique, rendering it a valuable complementary tool in clinical decision-making. Although tumour heterogeneity imposes challenges to the diagnostic accuracy of DW MRI concerning decision-making on regions-of-interest to be selected for data analysis, DW MRI offers valuable information as an ancillary diagnostic tool. This technique can avoid or delay a biopsy by pointing out benign characteristics of a tumour, thus supporting clinical imaging follow-up, or it can reinforce the need for a short-term biopsy if it suggests malignant characteristics of a tumour.

## Figures and Tables

**Table 1:** Patient characteristics of the study cohort.

Case #	Gender	Age at Diagnosis	Diagnosis	Tumour Location
1	M	7 y	GN	Posterior mediastinum
2	F	7 days	NB	Presacral
3	F	9 m	NB	Posterior mediastinum
4	M	12 m	NB	Presacral
5	M	8 y 3 m	GN	Retroperitoneum
6	M	32 m	GNB	Carotid bifurcation
7	M	3 y 8 m	NB	Retroperitoneum
8	M	3 m	NB	Retroperitoneum
9	M	9 y 8 m	GN	Presacral
10	F	9 m	NB	Paravertebral
11	F	5 y 7 m	GNB	Paravertebral
12	F	9 m	NB	Presacral
13	M	3 days	NB	Posterior mediastinum
14	F	15 y 11 m	GN	Suprarenal
15	F	12 y 1 m	GN	Suprarenal
16	M	6 y 4 m	GNB	Suprarenal
17	M	7 m	NB	Posterior mediastinum
18	F	17 m	NB	Posterior mediastinum
19	F	4 y	NB	Retroperitoneum
20	M	17 m	NB	Posterior mediastinum
21	M	5 m	NB	Suprarenal
22	M	13 m	NB	Suprarenal
23	F	13 m	GNB	Posterior mediastinum
24	M	9 y 10 m	NB	Suprarenal
25	M	3 y 11 m	NB	Suprarenal
26	F	3 y 8 m	NB	Presacral
27	M	11 y 7 m	GNB	Retroperitoneum
28	F	36 m	NB	Suprarenal
29	F	3 y 2 m	GN	Posterior mediastinum
30	F	1 m	NB	Posterior mediastinum
31	M	17 days	NB	Neck
32	F	2 y 4 m	NB	Paravertebral
33	F	4 y 4 m	GNB	Suprarenal
34	M	14 y 7 m	GN	Retroperitoneum
35	M	16 y 7 m	GN	Posterior mediastinum
36	F	6 y 11 m	GN	Retroperitoneum—intramuscular
37	F	1 y 9 m	NB	Posterior mediastinum
38	M	3 m	NB	Retroperitoneum
39	M	1 m 22 d	NB	Retroperitoneum—intramuscular
40	M	15 y 7 m	GN	Retroperitoneum
	Median (months)		34 (2 y 10 m)	
**Females = 18 (45%)**	Mean (months)		54.7 (4 y 6 m)	
	Min.		7 days	
	Max.		16 y 7 m	
	SD (months)		60.2 (5 y)	

Abbreviations: M, male; F, female; y, years; m, months; NB, neuroblastoma; GNB, ganglioneuroblastoma; GN, ganglioneuroma; SD, standard deviation.

**Table 2:** General tumour characteristics of the study cohort.

Case #	Diagnosis	International Neuroblastoma Risk Group (INGRSS)	MYCN Status	Pre-Treatment Risk Group	Multiple Body Compartments (Y/N)	Multi-Focal (Y/N)	Size of Tumour (AP Diam.) in mm	Size of Tumour (CC Diam.) in mm	Size of Tumour (TRV Diam.) in mm	Total Tumour Volume (cc)	Lobulated (Y/N)	Heterogenous (Y/N)	Calcification (Y/N)	Necrosis (Y/N)	Metastases (Y/N)	Metastases Type	Vascular Encasement (Y/N)	Vascular Encasement Vessels Involved	Adjacent Organ Infiltration (Y/N)	Type of Organ Infiltrated	Lymphadenopathy (Y/N)	Intraspinal Invasion (Y/N)	IDRF (Y/N)
1	GN	NA			N	N	37	43	28	23.32	N	Y	N	N	N		Y	Right vertebral artery	N		N	N	Y
2	NB	MS	Non-amplified	Low risk	N	N	42	65	39	55.75	Y	Y	N	N	N		N		Y	Left sacral bone	N	Y	Y
3	NB	MS	Non-amplified	Low risk	Y	N	35	69	49	61.96	Y	Y	N	N	Y	Right cutaneous neck	Y	Right subclavian artery, proximal vertebral artery	Y	Right posterior 4th rib	Y	Y	Y
4	NB	L1	Non-amplified	Low risk (surgery only)	N	N	65	57	53	102.11	Y	Y	Y	N	N		N		N		N	N	N
5	GN	NA			N	N	80	96	66	263.58	Y	Y	N	N	N		N		N		N	N	N
6	GNB	NA			N	N	16	32	23	6.17	N		N	N	N		N		N		N	N	Y
7	NB	M	Non-amplified	High risk	N	N	23	31	26	9.71	N	Y	N	N	Y	Bones	N		N		Y	N	N
8	NB	MS	Non-amplified	Low risk	N	N	63	61	56	111.91	Y	Y	N	N	Y	Liver, paraspinal, skull base, left orbital, left proximal femur	Y	Celiac, SMA	N		N	N	Y
9	GN	NA			N	N	36	60	40	44.93	Y	Y	N	N	N		N		N		N	N	N
10	NB	L1	Non-amplified	Low risk (surgery only)	N	N	13	18	8	0.98	Y	N	N	N	N		N		N		N	N	N
11	GNB	NA			N	N	34	64	26	29.42	Y	Y	N	N	N		Y	Thoracic aorta	N		N	N	Y
12	NB	L2	Non-amplified	Intermediate risk	N	N	74	115	97	432.22	Y	Y	Y	N	Y	Left paraaortic lymph nodes	N		N		Y	Y	Y
13	NB	L2	Non-amplified	Intermediate risk	N	N	47	52	50	63.98	Y	Y	Y	N	N		N		Y	Left paravertebral muscles	N	Y	Y
14	GN	NA			N	N	30	29	15	6.83	N	N	N	N	N		N		N		Y	N	N
15	GN	NA			N	N	86	80	47	168.15	Y	Y	Y	N	N		Y	IVC	N		N	N	Y
16	GNB	NA			N	N	33	30	30	15.44	Y	Y	Y	N	N		N		N		N	N	N
17	NB	L2	Non-amplified	Intermediate risk	N	N	56	93	92	249.15	Y	Y	Y	N	N		Y	Aorta	N		N	Y	Y
18	NB	L1	Non-amplified	Low risk (surgery only)	N	N	38	39	25	19.27	Y	Y	N	N	N		N		N		N	N	N
19	NB	L2	Non-amplified	Intermediate risk	N	N	83	127	95	520.73	Y	Y	N	Y	N		Y	Left common iliac artery, left renal artery and vein	Y	Left psoas muscle	N	N	Y
20	NB	L1	Non-amplified	Low risk (surgery only)	N	N	23	43	24	12.34	Y	N	N	N	N		N		N		N	N	N
21	NB	MS	Non-amplified	Low risk	N	N	43	57	58	73.92	Y	Y	N	N	Y	Liver	Y	Aorta, IVC, celiac, SMA, left renal artery and vein	N		N	N	Y
22	NB	L1	Non-amplified	Low risk (surgery only)	N	N	19	24	13	3.08	Y	Y	N	N	N		N		N		Y	N	N
23	GNB	NA			N	N	16	16	8	1.06	N	N	N	N	N		N		N		N	N	N
24	NB	M	Unknown	High risk	N	N	104	170	104	962.75	Y	Y	N	Y	Y	Liver, pelvic bones, sacrum, spine, left iliac bone	Y	SMA, left renal vessels	Y	Left kidney	N	N	Y
25	NB	M	Unknown	High risk	N	N	44	49	28	31.39	Y	Y	Y	N	Y	Liver, bones (right prox femur, left iliac bone, right sacrum, T12, L3 vertebral bodies)	N		N		Y	N	N
26	NB	L2	Non-amplified	Low risk	N	N	106	121	76	510.39	Y	Y	N	Y	N		N		N		N	Y	Y
27	GNB	NA			N	N	63	100	61	199.84	Y	Y	N	N	N		N		N		N	N	N
28	NB	M	Non-amplified	High risk	N	N	74	90	60	207.79	Y	Y	N	Y	Y	Vertebral bodies, pelvic bones	Y	Left renal artery and vein	N		N	N	N
29	GN	NA			N	N	11	22	7	0.88	N	N	N	N	N		N		N		N	N	N
30	NB	L2	Non-amplified	Intermediate risk	N	N	28	42	50	30.58	Y	Y	N	N	N		Y	Descending aorta	Y	Left paraspinal muscles	N	N	Y
31	NB	L2	Non-amplified	Low risk	N	N	22	38	24	10.43	Y	Y	N	N	N		Y	Right internal jugular vein	N		N	N	Y
32	NB	L1	Non-amplified	Low risk (surgery only)	N	N	34	17	24	7.26	Y	N	N	N	N		Y	Bilateral common iliac arteries	N		N	N	Y
33	GNB	NA			N	N	79	107	105	464.73	Y	Y	N	N	N		Y	Right renal artery and vein	N		N	N	Y
34	GN	NA			N	N	60	89	63	176.15	Y	Y	Y	Y	N		N		N		N	N	N
35	GN				N	N	24	30	30	11.31	N	N	N	N	N		N		N		N	N	N
36	GN	NA			N	N	32	60	50	50.27	N	Y	N	N	N		N		N		N	N	N
37	NB	L1	Non-amplified	Low risk (surgery only)	N	N	37	18	55	19.18	N	N	N	N	N		N		N		N	N	N
38	NB	L2	Non-amplified	Intermediate risk	N	N	26	41	42	23.44	Y	Y	Y	N	N		N		Y	Right paraspinal muscles	N	Y	Y
39	NB	L2	Non-amplified	Intermediate risk	N	N	37	48	30	27.9	Y	Y	N	N	N		N		Y	Right paraspinal muscles L2–3	N	Y	Y
40	GN	NA			N	N	47	86	63	133.33	Y	Y	N	N	N		N		N		N	N	N

Abbreviations: Y, yes; N, no; NA, not applicable; NB, neuroblastoma; GNB, ganglioneuroblastoma; GN, ganglioneuroma; IDRF, image-defined risk factor.

**Table 3:** Tumour characteristics of the study cohort on MR imaging.

Case #	Diagnosis	Tumour Location	T1	T2	Diffusion Restriction (Y/N)	b-Value (s/mm^2^)	Mean ADC Value	Whole Lesion (10^−3^ mm^2^/s)	Enhancement Type (Heterogenous, Homogenous, None)
1	GN	Posterior mediastinum	iso	hyper	Y	800	1484.07	1713.60	heterogenous
2	NB	Presacral	iso	intermediate	Y	800	643.17	677.70	heterogenous
3	NB	Posterior mediastinum	iso	hyper	Y	1000	325.33	360.30	Homogenous
4	NB	Presacral	iso	hyper	Y	600	781.07	932.80	Homogenous
5	GN	Retroperitoneum	hypo	hyper	Y	600	1752.20	2173.80	heterogenous
6	GNB	Carotid bifurcation	hypo	hyper	Y	1000	3650.60	3956.70	Homogenous
7	NB	Retroperitoneum	hypo	hyper	Y	600	383.47	475.40	No contrast
8	NB	Retroperitoneum	iso	hyper	Y	600	678.33	810.70	heterogenous
9	GN	Presacral	hypo	hyper	Y	600	2015.53	2385.10	heterogenous
10	NB	Paravertebral	iso	hyper	Y	800	1071.17	1048.50	homogenous
11	GNB	Paravertebral	hypo	hyper	Y	800	1581.13	1522.40	heterogenous
12	NB	Presacral	hypo	hyper	Y	600	849.87	1011.60	heterogenous
13	NB	Posterior mediastinum	hyper	hyper	Y	600	649.33	710.10	Homogenous
14	GN	Suprarenal	hypo	hyper	N	600	1125.53	1048.00	homogenous
15	GN	Suprarenal	hypo	hyper	Y	600	968.07	1020.80	heterogenous
16	GNB	Suprarenal	hypo	hyper	Y	600	810.80	814.10	heterogenous
17	NB	Posterior mediastinum	iso	hyper	Y	600	773.20	774.30	heterogenous
18	NB	Posterior mediastinum	hypo	hyper	Y	600	1080.87	1066.30	Homogenous
19	NB	Retroperitoneum	hypo	hyper	Y	800	1226.33	1421.50	heterogenous
20	NB	Posterior mediastinum	iso	hyper	Y	600	981.20	980.00	Homogenous
21	NB	Suprarenal	hypo	iso	Y	600	517.93	525.90	heterogenous
22	NB	Suprarenal	hypo	iso	Y	600	772.33	951.40	heterogenous
23	GNB	Posterior mediastinum	hypo	hyper	Y	600	1437.53	1442.40	Homogenous
24	NB	Suprarenal	hypo	hyper	Y	1000	649.00	1075.30	heterogenous
25	NB	Suprarenal	iso	hyper	Y	600	657.43	747.70	heterogenous
26	NB	Presacral	hypo	hyper	Y	600	1657.10	1897.00	heterogenous
27	GNB	Retroperitoneum	hypo	hyper	Y	500	1048.93	1073.00	heterogenous
28	NB	Suprarenal	hypo	hyper	Y	800	741.20	791.60	heterogenous
29	GN	Posterior mediastinum	hypo	hyper	Y	600	803.80	965.00	Homogenous
30	NB	Posterior mediastinum	iso	hyper	Y	600	862.63	791.70	heterogenous
31	NB	Neck	iso	iso	Y	1000	243.97	237.50	No contrast
32	NB	Paravertebral	hypo	hyper	Y	600	1200	1228.20	heterogenous
33	GNB	Suprarenal	iso	hyper	Y	600	736.07	763.70	heterogenous
34	GN	Retroperitoneum	hypo	hyper	N	800	1700.47	1853.20	heterogenous
35	GN	Posterior mediastinum	iso	hyper	N	800	1189.07	1126.20	Homogenous
36	GN	Retroperitoneum—intramuscular	iso	hyper	N	800	1577.77	1708.90	No contrast
37	NB	Posterior mediastinum	No	hyper	Y	600	1040	1000.00	Homogenous
38	NB	Retroperitoneum	iso	hyper	Y	1000	426.67	470.00	homogenous
39	NB	Retroperitoneum—intramuscular	iso	hyper	Y	800	706.33	835.70	heterogenous
40	GN	Retroperitoneum	hypo	hyper	Y	800	1475.33	2125.50	heterogenous

Abbreviations: Y, yes; N, no; NB, neuroblastoma; GNB, ganglioneuroblastoma; GN, ganglioneuroma; ADC, apparent diffusion coefficient; T1, T1-weighted; T2, T2-weighted.

**Table 4:** Descriptive statistics of quantitative features by tumour severity and diagnosis for neuroblastic tumours included in our retrospective study.

Severity	Variable	Mean	SD	LCLM 95%	UCLM 95%	Median	Minimum	Maximum
Benign (N = 16)	Total volume (cc)	155	266	31	168	37	1	1166
	ADC small	1.54	0.69	1.15	1.94	1.53	0.71	3.82
	ADC large	1.63	0.71	1.21	2.05	1.52	0.76	3.81
	Age at diagnosis (months)	101	1	75	139	96	14	193
Malignant (N = 24)	Total volume (cc)	147	234	49	246	43	1	956
	ADC small	0.85	0.38	0.68	0.95	0.78	0.20	1.92
	ADC large	0.91	0.71	0.73	1.03	0.88	0.76	1.94
	Age at diagnosis (months)	20	1	9	32	10	0	119
**Diagnosis**	**Variable**	**Mean**	**SD**	**LCLM 95%**	**UCLM 95%**	**Median**	**Minimum**	**Maximum**
GN (N = 10)	Total volume (cc)	93	90	23	153	48	1	264
	ADC small	1.52	0.44	1.22	1.83	1.63	0.88	2.16
	ADC large	1.64	0.51	1.24	2.04	1.72	0.95	2.40
	Age at diagnosis (months)	121	54	93	173	132	34	193
GNB (N = 6)	Total volume (cc)	269	430	75	314	22	1	1166
	ADC small	1.59	1.05	0.38	2.79	1.29	0.71	3.83
	ADC large	1.63	1.04	0.41	2.81	1.21	0.76	3.81
	Age at diagnosis (months)	63	40	18	110	58	14	140
NB (N = 24)	Volume	147	234	49	246	43	1	956
	ADC small	0.82	0.32	0.68	0.95	0.78	0.20	1.66
	ADC large	0.88	0.35	0.73	1.03	0.88	0.24	1.66
	Age at diagnosis (months)	21	26	9	32	10	0	119

Abbreviations: LCLM = lower confidence limit for the mean; UCLM = upper confidence limit for the mean; N = number of patients; cc = cubic centimetres; GN = ganglioneuroma; GNB = ganglioneuroblastoma; NB = neuroblastoma; SD = standard deviation; ADC = apparent diffusion coefficient.

**Table 5:** Descriptive imaging features of the study cohort diagnosed with neuroblastic tumours.

Imaging Features	NB (*n* = 24)		GNB (*n* = 6)		GN (*n* = 10)		*p*-Value
	Total No.	%	Total No.	%	Total No.	%	
Multifocal disease	0	0	0	0	0	0	-
Multiple body compartments	1 *	4.2	0	0	0	0	-
Lobulated (presence)	22	91.6	4	66.7	5	50	0.01
Heterogeneous (presence)	20	83.3	4	66.7	7	70	0.74
Calcifications (presence)	6	25	1	16.7	2	20	1.0
Necrosis (presence)	4	16.7	0	0	1	10	0.82
Predominant T1 signal							0.53
Hypointense	10	41.7	5	83.3	7	70	
Isointense	13	54.2	1	16.7	3	30	
Hyperintense	1	4.1	0	0	0	0	
Predominant T2 signal							0.83
Hypointense	0	0	0	0	0	0	
Isointense	4	16.7	0	0	0	0	
Hyperintense	20	83.3	6	100	10	100	
Contrast enhancement							1.0
Homogeneous	8	33.3	2	33.3	3	30	
Heterogeneous	14	58.3	4	66.7	6	60	
No contrast	2	8.3	0	0	1	10	
Image-defined risk factors (IDRFs)	15	62.5	2	33.3	2	20	0.07
Vascular encasement (presence)	10	41.7	2	33.3	2	20	0.53
Intraspinal invasion (presence)	8	33.3	0	0	0	0	0.04
Adjacent organ infiltration (presence)	8	33.3	0	0	0	0	0.04
Tracheal compression (presence)	3	12.5	0	0	0	0	0.72
Metastatic disease at diagnosis (presence)	8	33.3	0	0	0	0	0.04
Diffusion restriction (presence)	24	100	6	100	6	60	0.005

Abbreviations: NB = neuroblastoma; GNB = ganglioneuroblastoma; GN = ganglioneuroma. * Neck–chest.

**Table 6:** Diagnostic accuracy measures for quantitative MRI features and age at diagnosis based on neuroblastic tumours included in the retrospective study.

Feature	Sensitivity (95% CI)	Specificity (95% CI)	Positive LR (95% CI)	Accuracy (95% CI)	DOR and Log DOR
ADC cut-off small ROI, 1.06 × 10^−3^ mm^2^/s	0.83 (0.68, 0.98)	0.75 (0.54, 0.98)	3.33 (1.40, 7.94)	0.80 (0.64, 0.91)	DOR = 15.14 Log DOR = 2.71
ADC cut-off large ROI, 1.22 × 10^−3^ mm^2^/s	0.79 (0.63, 0.94)	0.83 (0.62, 1.00)	2.44 (1.28, 4.65)	0.80 (0.64, 0.91%)	DOR = 18.33 Log DOR = 2.91
Tumour volume cut-off, 56 cc	0.50 (0.29, 0.71)	0.63 (0.35, 0.85)	1.33 (0.63, 2.83)	0.55 (0.38, 0.71)	DOR = 1.67 Log DOR = 0.51
Age at diagnosis cut-off, 48 months	0.96 (0.79, 1.00)	0.81 (0.54, 0.96)	5.11 (1.84, 14.22)	0.90 (0.76, 0.97)	DOR = 99.66 Log DOR = 4.60

Abbreviations: DOR = diagnostic odds ratio; ADC = apparent diffusion coefficient; LR = likelihood ratio; Log = logarithmic (natural). Legend: All cut-off values were determined by Youden’s Index, and accuracy measures were calculated to 95% confidence intervals, *p* < 0.05.

**Table 7:** Summary results of single-variable logistic regression analysis of neuroblastic tumours with the probability modelled for ‘severity’ of tumour.

Variable	Likelihood Ratio ^1^	*p*-Value	Point Estimate ^2^	Odds Ratio	95% CI	*p*-Value
ADC small ROI	18.77	<0.0001	3.88	48.52	4.09, 575.36	0.002
ADC large ROI	16.10	<0.0001	3.07	21.58	2.63, 177.01	0.004
Tumour volume	0.61	0.44	0.001	1.001	1.00, 1.005	0.46
Age at diagnosis	25.40	<0.0001	0.05	1.05	1.02, 1.08	0.002

^1^ Testing of the global null hypothesis using the Chi-square method. ^2^ Analysis of maximum likelihood estimates. Abbreviations: CI = confidence interval; *p* = probability; ADC = apparent diffusion coefficient; ROI = region of interest.

## Data Availability

The data that support the findings of this study are available in the Supplementary Materials of this article.

## Supplementary Materials

The following supporting information can be downloaded at: https://www.mdpi.com/article/10.3390/jcm13226660/s1, Table S1: Clinical and qualitative imaging features of neuroblastic tumours included in our study cohort; Table S2: MRI parameters used for individual study participants; Table S3: Inter-rater agreement for apparent diffusion coefficient (ADC) diffusion-weighted (DW) MRI measurements according to region-of-interest reading methods.

## Abbreviations

GN = ganglioneuroma; GNB = ganglioneuroblastoma; NB = neuroblastoma; ADC = apparent diffusion coefficient; DWI = diffusion-weighted imaging; MRI = magnetic resonance imaging; ROI = region of interest.
