# Supplementary material for: Characterisation of Paediatric Neuroblastic Tumours by Quantitative Structural and Diffusion-Weighted MRI
Source: J Clin Med. 2024 Nov 6;13(22):6660. doi: 10.3390/jcm13226660 (PMC11594407; doi:10.3390/jcm13226660)
Supplement: Supplementary file 1 [file jcm-13-06660-s001.zip › jcm-3196216-supplementary.pdf]

Journal of
Clinical Medicine

MDPI

Table S1. Clinical and qualitative imaging features of neuroblastic tumours included in our study cohort.

**Variable**	**Number - Benign**	**Proportion**	**Number-Malignant**	**Proportion**	**Fisher's Exact Test (P-value)**
**Sex-**Male	9	22.5%	13	32.5%	0.25
**Sex**-Female	7	17.5%	11	27.5	
**Metastases-**Yes	16	40.0%	8	20.0%	0.01
**Metastases**-No	16	40.0%	0	00.0	
**Heterogeneous-**Hyperintense*	0	0.0%	1	2.5%	0.08
**Heterogeneous-**Hypointense*	12	30.0%	10	25.0%	
**Heterogeneous**-Isointense*	4	10.0%	13	32.5%	
**Contrast-**Yes	10	25.0%	14	35.0%	0.12
**Contrast**-No	5	12.5%	8	20.0%	
**No Contrast**	1	2.5%	2	5.0%	

Abbreviations: Mets=Metastases, Hetero=Heterogeneous, Contrast=Contrast Enhancement. *Hyperintense on T2-weighted imaging; hypointense on T1-weighted imaging; isointense on T1-weighted imaging (in relation to the adjacent musculature).

Table S2. MRI parameters used for individual study participants.

	MRI Scanner	Magnetic field strength (Tesla)	TR	TE	Bandwidth	Matrix	FOV (cm)	Slice thickness (mm)	B-values (s/mm²)
1	SIEMENS Avanto	1.5	6500	76	1628	192/150	No info	4	50, 400, 800
2	Philips Acheiva	1.5	3396.2	63.947	1800	124/120	25	5.25	0, 100, 600
3	Philips Acheiva	1.5	2957.2	63.947	1795	124/120	25	5.25	0, 100, 600
4	Philips Acheiva	1.5	2298.3	56.384	2774	180/180	45	5	0, 100, 600
5	Philips Acheiva	1.5	2827.9	60.077	2347	188/186	36	5	0, 100, 600
6	Philips Acheiva	1.5	3181.9	66.29	1763	152/148	30	5	0, 100, 600
7	SIEMENS Avanto	1.5	6400	75	1660	188/158	No info	5	NA
8	SIEMENS Avanto	1.5	8300	75	1630	192/162	No info	5	NA
9	SIEMENS Skyra	3	6000	48	2330	134/108	No info	5	NA
10	SIEMENS Skyra	3	6800	48	2330	134/108	No info	6	NA
11	Philips Acheiva	1.5	3438.9	50	2743	120/118	24	3	0, 1000
12	SIEMENS Avanto	1.5	4800	76	1860	192/150	No info	4.5	50, 400, 800
13	Philips Acheiva	3	2304.4	59.768	2169	184/180	32	5	0, 100, 600
14	Philips Acheiva	1.5	2333.5	82.635	1327	152/165	23	4	0, 100, 600
15	Philips Acheiva	1.5	1668.4	56.784	2697	124/124	37	7	0, 50, 500
16	Philips Acheiva	3	7419.3	68.74	2321	144/141	26	5	0, 200, 400, 600
17	SIEMENS Avanto	1.5	7200	84	1628	192/150	No info	5	50, 400, 800
18	Philips Acheiva	1.5	5495.5	88.683	1595	132/131	20	5	0, 1000
19	Philips Acheiva	1.5	1659.3	60.613	1766	96/92	19	4	0, 100, 600
20	Philips Acheiva	1.5	2292.6	54.281	2439	144/144	29	5	0, 100, 600
21	Philips Acheiva	1.5	2624.5	63.947	1800	124/120	25	5.25	0, 100, 600
22	SIEMENS Avanto	1.5	8923	124	1628	192/132	No info	4	NA
23	Philips Acheiva	1.5	2605.1	64.255	1800	124/120	25	5.25	0, 100, 600
24	Philips Acheiva	1.5	2592.6	64.255	1800	124/120	25	5.25	0, 100, 600

25	Philips Acheiva	1.5	1550.4	64.083	1786	128/124	26	5	0, 100, 600
26	Philips Acheiva	1.5	1525.3	63.828	1796	124/120	25	5	0, 100, 600
27	SIEMENS Skyra	3	5100	52	2440	128/78	No info	5	50, 800
28	Philips Acheiva	1.5	1862.5	63.828	1799	124/120	25	5	0, 100, 600
29	Philips Acheiva	3	3887.4	58.26	2568	124/122	25	5	0, 100, 600
30	Philips Acheiva	1.5	6097.4	61.066	1959	148/146	22	4	0, 100, 600
31	SIEMENS Skyra	3	6600	50	2440	128/78	No info	5	50, 1000
32	Philips Acheiva	1.5	4492.3	93.858	1331	188/206	28	4	0, 100, 600
33	Philips Acheiva	1.5	4459.3	83.762	1326	188/204	28	4	0, 100, 600
34	SIEMENS Skyra	3	7300	53	2330	134/108	No info	5	50, 800
35	GE Signa HDxt	1.5	5250	64.3	1953.1	160/128	26	5	NA
36	Philips Acheiva	1.5	3721.7	64.09	1339.2	100/100	15	5	0, 1000
37	SIEMENS Skyra	3	2300	52	2440	128/78	No info	5	0, 100, 600
38	Philips Acheiva dStream	1.5	3118.2	81.68	1185	132/146	20	4	0, 100, 600
39	Philips Acheiva dStream	1.5	2647.6	59.295	2098	96/92	30	2	0,1000
40	SIEMENS Skyra	3	8000	46	1925	104/72	No info	5	NA

Abbreviations: MRI=magnetic resonance imaging; FOV=field of view; TR=Repetition Time; TE=Time to Echo.

Table S3. Inter-rater agreement for apparent diffusion coefficient (ADC) diffusion-weighted (DW) MRI measurements according to region-of-interest reading methods.

**Feature**	**Reader 1**	**Reader 2**	**Agreement (%)**	**Rating by %**	**Kappa statistic (95% CI)**	**Kappa rating**
Average mean ADC small ROI (x10^{-6} mm^2/s)	1095	1214	86.78%	Strong	0.74 (0.72-0.76)	Substantial
Average median ADC small ROI (x10^{-6} mm^2/s)	967	1031	92.00%	Very Strong	0.84 (0.80-0.88)	Excellent
Average mean ADC large ROI (x10^{-6} mm^2/s)	1203	1238	93.86%	Very Strong	0.88 (0.87-0.89)	Excellent
Average median ADC large ROI (x10^{-6} mm^2/s)	1006	997	98.00%	Very Strong	0.96 (0.91-1.00)	Excellent
Average area (mm^2)	1211	1452	69.80%	Moderate	0.40 (0.38-0.42)	Fair agreement
SD (mm^2)	203	251	64.07%	Moderate	0.28 (0.26-0.30)	Fair agreement

Percent agreement rating scale: 40-59%=weak, 60-79%=moderate, 80-90%=strong, 90-100%=very strong
Kappa statistic rating scale:0=agreement equivalent to chance, 0.10-0.20=slight agreement, 0.21-0.40=fair agreement, 0.41-0.60=moderate agreement, 0.61-80=substantial agreement, 0.81-0.99=excellent agreement, 1=perfect agreement [[i]]

Abbreviations: ADC=apparent diffusion coefficient; ROI=region-of-interest; SD=standard deviation; CI=confidence interval.
